# The PPSQ: assessing parental, child, and partner’s playfulness in the preschool and early school years

**DOI:** 10.3389/fpsyg.2023.1274160

**Published:** 2023-12-04

**Authors:** Jean-François Bureau, Khachadour Bandk, Audrey-Ann Deneault, Jessica Turgeon, Harshita Seal, Patricia Brosseau-Liard

**Affiliations:** ^1^School of Psychology, University of Ottawa, Ottawa, ON, Canada; ^2^Département de psychologie, Université de Montréal, Montréal, QC, Canada; ^3^Département de Psychoéducation, Université du Québec en Outaouais, Gatineau, QC, Canada

**Keywords:** parental playfulness, child playfulness, co-parenting, self-report, early childhood

## Abstract

**Introduction:**

Developmental research has traditionally focused on parenting behaviors such as nurturance and care, due to a focus on mothers’ behaviors. Other parenting dimensions such as parental playfulness (i.e., use of creativity, imagination, and humor during parent–child interactions) have comparatively received little attention. Although some measures tap into parents’ and children’s playfulness, these measures are limited. Indeed, they do not assess multiple domains of playfulness (i.e., both parents’ and the child’s playfulness) or focus on one specific setting such as children’s play with peers. Additionally, existing measures do not consider parents’ reactions to their partners’ playfulness. To address this gap, we created the *Playful Parenting Style Questionnaire* (PPSQ), which assesses three domains of playfulness: (a) parental domain, (b) child domain, and (c) partner domain. The current study is part of a validation effort of the PPSQ using a quantitative design. We aimed to explore the structure of the PPSQ by conducting an exploratory factor analysis (EFA) for each domain of playfulness; and assess the construct validity of the PPSQ factors by examining the association between factors and existing measures of playful parenting, child playfulness, and co-parenting.

**Method:**

The sample includes 347 parents (294 mothers and 53 fathers) of preschool/school-age children (*M* = 5.10 years; 182 girls, 127 boys). Parents were mostly White (76%) and from a low socioeconomic risk background. Parents completed a series of online questionnaires including the PPSQ, 3 existing measures of parent playfulness (Parental Playfulness Questionnaire; Adult Playfulness Scale; Challenging Parenting Behavior Scale), 2 existing measures of child playfulness (Child Behavior Inventory; Children’s Playfulness Scale), a coparenting instrument (Co-parenting Relationship Scale), and sociodemographic information.

**Results:**

The EFA revealed 4 factors for parental playfulness, 1 factor for child playfulness, and 3 factors for partner’s playfulness. The construct validity analyses identified multiple associations indicating convergence with existing measures for the parent and partners domain but not the child factor.

**Discussion:**

This study allowed for a better understanding of the playful dynamics that occur within a family.

## Introduction

Most theoretical models underscore the influence of child–mother relationships on children’s development. However, societal changes in Western countries over the past decades (e.g., women’s increased participation in the workforce) have led to increased paternal involvement in childcare (Cabrera et al., 2000; Sayer, 2018), especially during the preschool years (i.e., 3 to 5 years old; Lamb, 2004). Consequently, researchers have made multiple calls for the inclusion of fathers in developmental science, particularly in conjunction with a family systems approach that includes both parents (Cowan, 1997; Cabrera et al., 2014; Fagan et al., 2014). In line with these recommendations, there has been a proliferation of research on the role that fathers play in children’s development (e.g., Dagan et al., 2021; Deneault et al., 2022). Recent research including both fathers and mothers has generally shown that the constructs of fathering and mothering (e.g., sensitivity, playfulness, challenging parenting behaviors) are more similar than was previously expected (Fagan et al., 2014; Deneault et al., 2022). Fagan et al. (2014) therefore suggest moving away from conceptualizing mothering and fathering differently to instead focus on the various types of behaviors, skills, and attributes that both parents can display, regardless of gender (Cabrera et al., 2017). In line with this suggestion, the current study aims to explore parental playfulness in mothers and fathers along with their perception of child playfulness and their partner’s reaction to their own playfulness.

One aspect of parenting that has been extensively studied is parental sensitivity, that is the parent’s ability to respond to children’s needs in an efficient and timely manner (Ainsworth, 1969). One reason why sensitivity has received considerable attention is its hypothesized key role in fostering secure child–parent attachment (Ainsworth et al., 1978). Meta-analytic work suggests that, although sensitivity is robustly associated with attachment security, this association is only moderate in magnitude. This suggests that other parental behaviors may also foster positive child–parent relationships (van IJzendoorn and de Wolff, 1997; Lucassen et al., 2011; Verhage et al., 2016). As a result, many researchers have sought to expand the range of parental factors that are commonly studied as contributors to the quality of the child–parent relationship. For example, recent research explored the impact of parents’ challenging behaviors (StGeorge and Freeman, 2017), parents’ autonomy allowance (Olofson and Schoppe-Sullivan, 2022), parents’ attribution regarding child inner world (McMahon and Bernier, 2017), parents’ sense of self-efficacy (Albanese et al., 2018), parents’ engagement (Volling and Palkovitz, 2021), and the quality of the co-parenting relationship (Brown et al., 2010) on the parent–child relationship.

In recent years, some scholars have dedicated their efforts to study another frequently neglected aspect of parental behaviors, that is parental playfulness (Cabrera and Roggman, 2017). Parental playfulness is defined as a parent’s spontaneous use of physical, cognitive, and social behavior marked with creativity, joy, imagination, and humor during child–parent interactions (Cabrera et al., 2017; Menashe-Grinberg and Atzaba-Poria, 2017). Playfulness is characterized by positive affect (e.g., shared enjoyment, genuine laughter) together with a cognitive component (e.g., creativity, perspective-taking). A playful parent thinks flexibly, takes risks with ideas, and allows for creative thoughts to emerge (Atzaba-Poria, 2018).

The preschool years are a developmental period particularly well-suited to the use of parental playfulness (Amodia-Bidakowska et al., 2020), as this period is marked by an increase in children’s cognitive (e.g., theory of mind), social (e.g., interactions outside of the family unit), and linguistic abilities. These developmental changes may influence which activities child–parent dyads engage in. For example, Lamb (2004) suggests that fathers engage in less physical play and use more cognitively demanding behaviors during child-father interactions. When anticipating their child’s entry to school, it is possible that fathers, like mothers, put less emphasis on physically challenging interactions, such as being able to win a wrestling match, and instead attempt to challenge their child cognitively. Playful parents may do so by proposing unconventional use of toys or using surprising scenarios in symbolic play (Labrell, 1994). Parents’ playful behaviors contradict the traditional ways of smoothly behaving (e.g., avoiding upsetting the child or proposing predictable conventional themes in play). Some evidence suggests that forcing children to process/adapt to such irregularities may be as important as routines and regularities in improving their cognitive development. As suggested by Labrell (1996), it is possible that parents complement each other by offering both conventional and unconventional behaviors in different contexts (e.g., pretend play, conversation).

In addition to stimulating cognitive development, parental playfulness may foster a secure attachment relationship. Indeed, Bowlby (1982) stated that smiling, laughing, and expressing joy are all important behaviors signaling that an individual (i.e., the child or the parent) is interested in interacting with the partner, thereby contributing to the establishment of a securely attached relationship. Over the years, these positive parental behaviors have been somewhat neglected by attachment researchers. This is despite empirical evidence that parental playfulness showed significant associations with attachment-related concepts such as parental scaffolding and sensitivity (Aldoney and Prieto, 2021). Parental playfulness is also associated with various aspects of children’s social–emotional adaptation, such as lower anxiety (Majdandžić et al., 2018), better emotion regulation skills (Cabrera et al., 2017; Shorer et al., 2021), less behavioral problems (Levavi et al., 2020), lower negativity (Menashe-Grinberg and Atzaba-Poria, 2017), and increased vocabulary skills (Cabrera et al., 2017). Importantly, further empirical evidence also suggests this parental playfulness can be displayed by mothers and fathers (Bureau et al., 2014; Menashe-Grinberg and Atzaba-Poria, 2017).

### Assessing parental playfulness

There are only a few instruments that measure parental playfulness, either through observation or self-report. Atzaba-Poria et al. (2014) developed the Parental Playfulness Scale (PPS), an observational instrument assessing both playfulness and creativity. This scale has been used in multiple studies with infants (Atzaba-Poria et al., 2014; Cabrera et al., 2017; Levavi et al., 2020; Aldoney and Prieto, 2021; McDorman et al., 2021; Roy and Kumar, 2022; Léniz-Maturana et al., 2023), which identified moderate associations between parental playfulness and various child–parent outcomes. Despite the clear usefulness of this measure, observing parents in a playful setting with their child requires a considerable amount of resources, and playfulness in particular can be hard to elicit in a laboratory setting. Developing self-reported measures as a complementary source of information is therefore important, as these measures could help overcome these limitations and be used in studies without an observational component.

A few questionnaire measures exist to assess different aspects of parental playfulness. Majdandžić et al.’s (2018) Challenging Parenting Behavior Questionnaire (CPBQ) is one such example, which includes subscales such as teasing, rough-and-tumble play (RTP), and encouragement of risk taking, all of which may also be indicative of parental playfulness. The authors found similar factor structures for both mothers and fathers from two countries (the Netherlands and Australia). The parental playfulness subscales (i.e., teasing, risk-taking, and RTP) were negatively associated with child anxiety only in the Australian sample. Another questionnaire is the one of Shorer et al. (2021) which focuses specifically on parental playfulness. This questionnaire, however, is relatively brief (20 questions) and only provides a total playfulness score. Although the psychometric properties of the instrument have not yet been published, the authors found a positive association between parental playfulness and children’s emotion regulation skills.

Another questionnaire measure of playfulness is the Adult Playfulness Scale (APS; Glynn and Webster, 1992), which is not, however, specific to playfulness within the parenting context. This scale assesses adult playfulness as a personality trait and was explored in relation with various dimensions of workplace functioning. Nonetheless, it is also important to understand playfulness specifically within the context of child–parent relationships, which create frequent opportunities to display playfulness, even for someone who may have low scores on trait playfulness. As such, it is important to build upon these self-report assessments to design a measure that specifically assesses parental playfulness, while measuring various dimensions of it. These dimensions would allow for a more comprehensive investigation of the complex dynamics of child–parent interactions, and to examine whether different aspects of playfulness relate to various outcomes related to the child and/or the child–parent relationship.

### Assessing child playfulness and reaction to partner’s playfulness

While the playfulness of interactions between young children and their parents may depend more upon the parent’s behaviors and attitudes, it is possible that children contribute to the playfulness of interactions as they grow older and become engaged members of the child–parent dyad. That is, children become able to express their goals and needs verbally and to adjust to their parent’s goals and motivation (Bowlby, 1982). This may translate into more playful interactions, as suggested by a recent systematic review of 78 studies (Amodia-Bidakowska et al., 2020), which showed that playful child-father interactions significantly increase during the preschool years. It is possible that parents use the child’s feedback during interactions to adjust their own behaviors and expectations. In this context, it becomes important to consider children’s playfulness when assessing the dyad’s playful dynamic during the preschool years. Pioneer work aiming to assess child playfulness includes observational assessment of children playing with peers (e.g., Rubin, 1977). A few questionnaires were also developed to assess child playfulness (e.g., Barnett, 1991; Rogers et al., 1998; Trevlas et al., 2003), usually designed to be reported by teachers, and therefore based on interactions with peers. However, child playfulness can be expressed in other settings than while interacting with peers. For example, a child may show a playful attitude when telling jokes during a family trip, or when teasing a sibling during a family meal. In line with our conceptualization of parental playfulness, in the current study we are interested in child playfulness as a personality trait that can be shown in a variety of contexts. Therefore, it was important to develop an assessment tool that goes beyond the sole focus of child playfulness while playing with peers.

Currently, no questionnaire encompasses both parents’ own and children’s playfulness within the same questionnaire, although these two dimensions are probably intertwined. Similarly, current measures do not account for one’s perception of their partner’s playfulness. We believe it is a crucial dimension to assess as playful parenting is unconventional and disruptive by nature, which may be frowned upon and not accepted by a co-parent. The partner’s disapproval may not only reduce parental involvement in playful behaviors (McBride et al., 2005; Andersen et al., 2017), but may also negatively influence its contributions to child socioemotional development (Morris et al., 2007; Marvin and Britner, 2008). For example, a parent who disapproves of a game initiated by their partner may criticize the partner in front of the child and put an end to the interaction. This will not only prevent the child from benefiting from a playful and joyful interaction, but it will also expose the child to parental conflict. Such a phenomenon has been documented widely in families where mothers interfere with their partner’s involvement (maternal gatekeeping; Gaunt, 2008), which can have a negative effect on the child’s development. On the contrary, a parent who approves of playful interactions between their partner and their child, regardless of their own playfulness, is more likely to be supportive, which should, in turn, be beneficial for the child.

### Current study

As a first step toward a more exhaustive assessment of the child–parent playful dynamic in the preschool years, the current study aims to validate a new playful parenting questionnaire: the *Playful Parenting Style Questionnaire* (PPSQ; Bureau et al., 2019). The questionnaire includes three sections: (1) parental playfulness, (2) child playfulness, and (3) the perception and reaction to the partner’s playfulness. The original set of questions included 85 items distributed among the three dimensions (41 for parent, 21 for child and 23 for partner). The items for parental playfulness and child playfulness were inspired by findings from a previous research project that included observational assessments of parent–child playful interactions (Bureau et al., 2014, 2017) as well as existing questionnaires (e.g., Rogers et al., 1998; Shorer et al., 2021) and observational assessments (e.g., Atzaba-Poria et al., 2014). For the partner dimension, due to the lack of previous research addressing this domain, we brainstormed and generated items based on our research experience. This original set of items included both positive and negative statements regarding each assessed dimension. The creation of the PPSQ aims to address the need for a comprehensive measure of playfulness across multiple members of the family. Previous research on playfulness generally focuses either on one parent only or the child in a specific setting (i.e., physical play with peers). However, we know, from a family system point of view (Kerr, 1981), the importance of considering the different dynamics occurring in a family setting. Therefore, we hope that the PPSQ could help explore how the perceptions of one’s own playfulness may interact with the perception of the partner’s playfulness in prediction of child development.

This project also contributes to the literature through its focus on the preschool period. Although the literature generally focuses on physical stimulation and play during infancy, the preschool years are an important period for playfulness given fathers’ great involvement in child rearing and more reciprocal partnerships between child and parents. Lastly, the current study also includes mothers and fathers, thereby contributing to the study of playfulness in parents of multiple genders. Due to the COVID-19 pandemic, this study had to be conducted online. Unfortunately, this prevented the collection of additional observational data on child–parent interactions or child outcomes.

## Objectives and hypotheses

This research project had two main objectives. The first objective was to explore the factor structure of a new playfulness questionnaire aiming to cover three aspects of playfulness, that is parental playfulness, child playfulness, and the reaction to and satisfaction with the partner’s playfulness (i.e., Playful Parenting Style Questionnaire; PPSQ).

The second objective sought to examine the construct validity of the PPSQ by first examining the associations between the factors for each section of the PPSQ and similar constructs assessed with existing questionnaires, when available (convergent validity). We assessed the convergent validity of the parental playfulness section with playfulness as measured in the Parental Playfulness Questionnaire (PPQ, Shorer et al., 2021), the Adult Playfulness Scale (APS, Glynn and Webster, 1992), as well as the teasing, rough-and-tumble play, and challenging parental behavior subscales of the Challenging Parenting Behavior Questionnaire (CPBQ, Majdandžić et al., 2018). We expected to find strong associations between factors of the PPSQ and similar constructs on the existing questionnaires (e.g., a rough-and-tumble PPSQ factor would be associated with the CPBQ). We also expected to find predictive validity in the form of moderate associations between different constructs that should nonetheless be related (e.g., a parental teasing PPSQ factor would be associated with the RTP subscale of the CPBQ).

Concerning the child domain, we assessed the convergent validity with child playfulness (Child Behaviors Inventory of Playfulness: CBI, Rogers et al., 1998), physical, social, and cognitive spontaneity (Children’s Playfulness Scale: CPS, Barnett, 1991), as well as joy (CPS) and sense of humor (CPS). We expected to find strong associations between factors of the PPSQ and these questionnaires if they assessed similar constructs. We also expected to find predictive validity in the form of moderate associations for different constructs that should nonetheless present an association (e.g., child rigidity on the PPSQ would be associated with child spontaneity assessed through the CPS).

Lastly, as no other questionnaires assess perception of partner’s playfulness, we were not able to assess convergent validity with existing questionnaires. However, we expected that the emerging factors should be related to dimensions of coparenting as assessed with the Coparenting Relationship Scales (CRS, Feinberg et al., 2012). More precisely, we expected that a positive perception of the partner’s playfulness would be associated with a positive view of the coparenting relationship.

## Method

### Participants

The participants, recruited throughout Canada over a 2-year period (2020–2022), consisted of 347 parents (*M_age_* = 35.74 years, *SD* = 5.09, Range = 24–47; 294 mothers and 53 fathers) of preschool children aged between 3 and 8 years old (182 girls, 127 boys, and 38 undisclosed; *M_age_* = 5.10 years, *SD* = 1.37, Range = 3–8). The inclusion criteria for the study were that the participant had to (a) speak either English or French and that (b) only 1 parent per family could answer the questionnaires. Parents from same-sex families were invited to participate, however, none participated. Non-biological parents were eligible to participate if they had lived with the child for at least two years and were a parental figure. Most parents were part of two-parent families (*n* = 303), while the rest were part of single-parent families (*n* = 44).

Participants were recruited through social media announcements, parenting forums, classified ads, ads on campus or in community centers, snowball technique, and the School of Psychology’s Integrated System of Research Participation (ISPR). Participants received a 10$ gift card as a compensation for their time. For participants recruited through the ISRP, in line with the program’s policy, participants were awarded one point toward their final grade in an introduction to psychology course (University of Ottawa). Most participants identified as White (*n* = 266), while the remaining participants identified as Asian (*n* = 27), Middle Eastern (*n* = 8), Black (*n* = 21), mixed ethnicity (*n* = 11), Latinx (*n* = 10 participants), Indigenous/First Nations/Métis (*n* = 1), or another ethnicity not listed (*n* = 4). Most of the participants identified English as their mother tongue (*n* = 182), whereas the remaining either identified French (*n* = 97), both English and French (*n* = 12), or another language (*n* = 57) as their mother tongue. Most of the participants stated that their occupation was working (*n* = 241), while the rest identified that they were studying (*n* = 31), at home (*n* = 41), unemployed (*n* = 4), temporarily at home due to Covid (*n* = 25), or other (*n* = 1). Most participants had mid-to-high income, with participants indicating that their family’s gross annual income (before tax and deductions) was either less than $20,000 (*n* = 11), between $20,000 and $50,000 (*n* = 39), between $50,000 and $100,000 (*n* = 93), or above $100,000 (*n* = 198). With respect to education, *n* = 33 participants indicated that their highest diploma was a high school degree, *n* = 69 a college degree, *n* = 149 an undergraduate degree, and *n* = 96 a graduate degree.

### Procedure

Participants registered via the ISPR to participate in the study, or they contacted the study team to receive an anonymous participation link. The contact with our team (vs. providing a link on the study advertisement) was done to exclude computerized auto-robots (Storozuk et al., 2020). After completing a consent form, participants completed a series of online questionnaires using Qualtrics, a secure website used to collect survey data. The Ethics Board of the University of (Ottawa) approved the procedure used in this study.

### Instruments

All questionnaires were translated from English to French and retranslated from French to English by an independent person with an expertise in translation. The double-blind, independent translation thus ensured the consistency of questionnaires across languages.

*The Playful Parenting Style Questionnaire* (*PPSQ:* Bureau et al., 2019). The PPSQ is a comprehensive questionnaire, answered by parents, evaluating parent and child playfulness, as well as the parent’s perception of their partner’s reaction to their own playfulness. Parents were asked to rate statements about interactions with their child and their partners on a 6-point Likert scale ranging from 1 (almost never) to 6 (very often). The original scale includes 41 items for parent playfulness, 21 items for child playfulness, and 23 items for partner’s playfulness. An example of a statement targeting parent playfulness is: “I tease my child.” A list of the 28 items retained after the exploratory factor analysis (EFA) for parent playfulness is presented in Table 1. An example of a statement assessing child playfulness is “My child easily gets offended or hurt by jokes.” A list of the 4 items retained after the EFA for child playfulness is presented in Table 2. An example of a statement evaluating partner playfulness is “I trust my partner’s ability to entertain our child.” A list of the 13 items retained after the EFA for partner playfulness is presented in Table 3. Among the retained items after the EFA, 5 items were reverse-scored.

**Table 1:** Exploratory factor analysis with varimax rotation for the parent domain section of the PPSQ.

Parent dimension	Fact. 1^a^	Fact. 2^b^	Fact. 3^c^	Fact. 4^d^
I suggest mixing elements from different games (e.g., building a Lego house for stuffed animals) when playing with my child.	**0.69**	0.01	0.29	0.23
I have fun with my child.	**0.67**	0.20	0.02	0.25
I am confident in my ability to entertain my child.	**0.62**	0.10	0.00	0.16
I build things with my child (e.g., playing with Lego blocks, building a fort, or building a snowman).	**0.62**	−0.02	0.04	0.23
I play tag or run after my child	**0.59**	0.28	0.18	0.49
I could make more of an effort when playing with my child.	**−0.58**	−0.21	0.21	−0.22
I act silly (e.g., clowning around, dancing, making funny faces) to make my child laugh.	**0.58**	0.31	0.15	0.33
I feel like I should play more often with my child.	**−0.57**	−0.21	0.16	−0.21
When playing with my child, I suggest unconventional uses for his/her toys (e.g., using blocks as dominoes, or using a ball as a planet for his/her figurines).	**0.56**	0.11	0.33	0.24
I tell jokes or funny stories to make my child laugh.	**0.56**	0.37	0.11	0.29
My child and I have fun without toys.	**0.54**	0.09	0.01	0.17
I think my child has fun when playing with me.	**0.53**	0.10	−0.12	0.09
I enjoy playing with my child.	**0.52**	0.15	−0.01	0.23
I like to laugh with my child	**0.52**	0.18	−0.02	0.15
When playing, I encourage my child’s initiatives.	**0.51**	−0.01	−0.13	0.06
I tease my child.	0.16	**0.77**	0.14	0.29
I play tricks on my child.	0.15	**0.70**	0.37	0.38
I am sarcastic when I interact with my child.	0.12	**0.65**	0.29	0.24
I tickle my child.	0.42	**0.58**	0.04	0.45
I answer my child’s questions with a joke.	0.14	**0.57**	0.42	0.27
I voluntarily break a rule in the presence of my child.	−0.06	0.22	**0.69**	0.26
I break the rules of a game and explain to my child that it’s okay.	0.05	0.16	**0.63**	0.17
I break the rules to entertain my child.	−0.05	0.29	**0.61**	0.23
I give my child false information, and then explain to him/her that it was a joke.	−0.01	0.43	**0.55**	0.19
My child and I have fun doing what would be considered impolite activities (e.g., farting contests).	0.17	0.23	**0.53**	0.35
I engage in roughhousing (e.g., play fighting) with my child.	0.09	0.24	0.32	**0.77**
I engage in play-fighting with my child or lift him/her up in the air.	0.36	0.41	0.16	**0.69**
I have pillow fights with my child.	0.26	0.24	0.24	**0.57**
Eigenvalue	9.29	5.35	3.32	2.82
% of Variance	23.76	13.70	8.49	7.21

a Factor 1 = Playful effort; ^b^Factor 2 = Teasing; ^c^Factor 3 = Rule defiance; ^d^Factor 4 = RTP.

Extraction method: principal axis factoring. Rotation method: promax. Rotation converged in 19 iterations.

**Table 2:** Exploratory factor analysis with varimax rotation for the child domain section of the PPSQ.

Child dimension	Factor 1^a^
My child gets offended when a story that he/she knows well gets changed.	**0.66**
My child gets angry when he/she does not understand a game.	**0.65**
My child easily gets offended or hurt by jokes.	**0.55**
My child has difficulty understanding when he/she is being teased or is the subject of a joke.	**0.52**
Eigenvalue	21.06
% of Variance	51.66

a Factor 1 = Rigidity/offended.

Extraction method: principal axis factoring. Rotation method: promax. Rotation converged in 8 iterations.

**Table 3:** Exploratory factor analysis with varimax rotation for partner domain section of the PPSQ.

Partner Dimension	Fact. 1^a^	Fact. 2^b^	Fact. 3^c^
I feel that my partner does not make enough of an effort when he/she plays with our child.	**0.88**	0.38	0.08
I feel that my partner does not play enough with our child.	**0.83**	0.38	0.01
I feel that my partner lacks energy when he/she plays with our child.	**0.75**	0.24	0.06
I trust my partner’s ability to entertain our child.	**−0.75**	−0.35	−0.16
I feel that my partner is not entertaining for our child.	**0.73**	0.36	0.11
I think my partner is good at entertaining our child.	**−0.71**	−0.30	−0.14
I think that my partner is funny	**−0.46**	−0.17	−0.18
I feel that my partner is too reckless when he/she plays with our child.	0.31	**0.83**	0.39
When my partner plays with our child, I’m afraid that my child will get hurt.	0.26	**0.78**	0.43
When my partner plays with our child, I feel that he/she goes too far.	0.33	**0.74**	0.31
My partner goes too far when he/she makes fun of our child.	0.35	**0.60**	0.28
I feel excluded from games that my partner and our child play.	0.17	0.43	**0.72**
I would like my partner to invite me to play with our child.	0.12	0.30	**0.66**
Eigenvalue	8.21	3.48	1.90
% of Variance	41.25	17.50	9.56

a Factor 1 = Partner’s lack of effort; ^b^Factor 2 = Partner’s Recklessness; ^c^Factor 3 = Feeling of exclusion.

Extraction Method: Principal Axis Factoring. Rotation Method: Promax. Rotation converged in 5 iterations.

*The challenging parenting behavior scale for parents of 4- to 6-Year-Old Children* (*CPBQ4-6*; Majdandžić et al., 2016, 2018). The CPBQ4-6 is a comprehensive self-report instrument that assesses the extent to which the parent encourages the child socio-emotionally and physically to exhibit risky behavior, or behavior that causes the child to go outside of their comfort zone. The original scale includes 39 items and 6 subscales: teasing, rough-and-tumble play, encouragement of risk taking, social daring, competition, and modeling. In addition to the subscales, a total score can be derived for an overall measure of CPBQ. Parents were asked to rate statements about interactions with their child on a 5-point Likert scale from 1 (not applicable) to 5 (completely applicable). An example of a statement under the teasing subscale is: “I play little tricks on my child.” For this study, only 3 subscales (18 items) were used: teasing, rough-and-tumble play, and encouragement of risk taking. Two items were reverse-scored. The questionnaire presents good reliability and validity (Majdandžić et al., 2016).

*The Parental Playfulness Questionnaire* (*PPQ*; Shorer et al., 2021). The PPQ is a comprehensive self-report scale that assesses parental playfulness in different every-day parent–child interactions. The PPQ consists of 20 items. Each item is rated on a 5-point Likert scale from 1 (totally disagree) to 5 (totally agree). An example of a statement under the teasing subscale is: “When my child refuses to eat a new kind of food, I look for creative ways to persuade him/her to give it a try” (Shorer et al., 2021). The total score is obtained by calculating the average of all 20 items, resulting in a total score ranging from 1 to 5. A higher score reflects greater parental playfulness. Seven items were reverse-scored. The questionnaire presents good reliability (Shorer et al., 2021).

*The Adult Playfulness Scale* (*APS*; Glynn and Webster, 1992). The APS is a comprehensive self-report scale that assesses adults’ trait playfulness whilst incorporating the factors of spontaneity, expressiveness, fun, creativity, and silliness that may influence organizational outcomes, especially in the workplace. The APS consists of 32 pairs of items. Each item is represented by an adjective dichotomy such as “calm-agitated” or childlike-mature.” Each item is rated on a 7-point Likert scale from 1 to 7, where 1 represents relatability to one end of the spectrum for the adjective dichotomy, and 7 represents relatability to the other end of the spectrum. All items, except two, are reverse-scored. The score for each item is averaged in order to calculate a total playfulness score. The questionnaire presents good reliability and validity (Glynn and Webster, 1992).

*The Child Behavior Inventory for Playfulness* (*CBI*; Rogers et al., 1998). The CBI is a comprehensive self-report instrument used by parents or teachers to assess a child’s playfulness as a trait characteristic. The CBI consists of 30 items. It is separated into a playfulness subscale and an externality subscale. The playfulness subscale contains 21 items that measures overall playfulness, orientation to a task, intrinsic motivation, non-linearity, freedom from externally imposed rules, and active involvement during the task. The externality subscale contains 7 items that measures behaviors likely to reduce a child’s ability to play. Two items, “Has a sense of humor” and “Displays exuberance much of the time” are thought to relate to playfulness but have been subject to proper validation for inclusion in the playfulness scale. For this reason, neither item was included in the playfulness subscale. The score of each item is summed to calculate a total score of playfulness, with higher scores representing higher playfulness. Example statements of the questionnaire are “Uses things in their own way” and “pretends a lot.” Each item is rated on a 5-point Likert scale from 1 (very uncharacteristic) to 5 (very characteristic). The questionnaire presents good reliability and validity (Rogers et al., 1998).

*The Children’s Playfulness Scale* (*CPS*; Barnett, 1991). The CPS is a comprehensive self-report instrument used by parents or teachers to assess children’s play style and play behavior. The CPS consists of 23 items. The questionnaire is separated into 5 different subscales: physical spontaneity, social spontaneity, cognitive spontaneity, manifest joy, and sense of humor. Each subscale either contains 4 or 5 items. Each item is rated on a 5-point Likert scale from 1 (sounds exactly like the child) to 5 (does not sound at all like the child). Two items were reverse-scored. Example statements of the questionnaires are: “the child initiates play with others” and “the child expresses enjoyment during play.” The questionnaire has good reliability and validity (Barnett, 1991).

*The Co-parenting Relationship Scale* (*CRS*; Feinberg et al., 2012). The CRS is a comprehensive self-report instrument that assesses the quality of co-parenting within a family (i.e., how two individuals function together in their parenting roles via supporting one another whilst sharing the responsibilities toward the children they care for). The CRS consists of 35 items divided into 7 subscales: Co-parenting Agreement, Co-parenting Closeness, Exposure to Conflict, Co-parenting Support, Co-parenting Undermining, Endorse Partner Parenting, and Division of Labor. Items are rated on a 6-point Likert scale rating either from 0 (not true of us) to 6 (very true of us) for the first 30 items; and 1 (never) to 5 (several times a day) for the final 5 items. An example statement from the Coparenting Agreement subscale is “My partner and I have the same goals for our child.” The questionnaire presents good reliability and validity (Feinberg et al., 2012).

## Analytic plan

For the first objective, we conducted separate exploratory factor analyses for each domain (parent, child, partner) to test the PPSQ dimensions. This analysis was done to refine items, reduce the number of items, and determine latent factors. A Promax rotation was used to take into account possible correlation between factors. The extraction method was a Principal Axis Factoring. We retained factors based on visual inspection of the scree plot. Prior to testing research objective 2, we explored potential covariates as well as the correlations between the different factors of the PPSQ. Then, we ran correlations or partial correlations between the obtained factors on the PPSQ and other variables of interest. As a follow-up to these correlational analyses, we ran multiple regression analyses to determine which variables of interest contribute significantly to the prediction of each factor of the PPSQ when shared variance is considered. Due to the number of analyses, we set a value of *p* of 0.01 as a cutoff to determine individual correlation significance.

## Results

### Descriptive data

In this section, we present the descriptive data (mean, SD and range) for the questionnaires used to assess the validity of the PPSQ (the descriptives of the PPSQ are presented in the next section). For the parental domain, we used the: PPQ total score (*M* = 3.62, *SD* = 0.45, ranging from 2 to 5); APS total score (*M* = 4.38, *SD* = 0.73, ranging from 2 to 6); CBPQ teasing score (*M* = 3.68, *SD* = 1.22, ranging from 1 to 5); CBPQ RTP score (*M* = 2.76, *SD* = 0.83, ranging from 1 to 5); and CBPQ Challenge to risk score (*M* = 3.81, *SD* = 0.73, ranging from 1 to 5). For the child domain, we used the: CBI total score (*M* = 4.04, *SD* = 0.52, ranging from 3 to 5); CPS physical spontaneity score (*M* = 4.16, *SD* = 0.65, ranging from 2 to 5); CPS social spontaneity score (*M* = 3.80, *SD* = 0.70, ranging from 2 to 5); CPS cognitive spontaneity score (*M* = 3.69, *SD* = 0.63, ranging from 1 to 5); CPS manifestation of joy score (*M* = 4.44, *SD* = 0.51, ranging from 3 to 5); and CPS sense of humor score (*M* = 3.79, *SD* = 0.75, ranging from 2 to 5). For the partner domain, we used the: CRS total score (*M* = 4.00, *SD* = 0.67, ranging from 0 to 6); CRS agreement score (*M* = 4.11, *SD* = 1.09, ranging from 0 to 6); CRS closeness score (*M* = 4.18, *SD* = 0.88, ranging from 0 to 6); CRS support score (*M* = 4.33, *SD* = 0.83, ranging from 0 to 6); CRS undermining score (*M* = 2.72, *SD* = 1.73, ranging from 0 to 6); CRS partner parenting score (*M* = 4.05, *SD* = 0.69, ranging from 0 to 6); and CRS division of labor score (*M* = 3.60, *SD* = 1.53, ranging from 0 to 6).

### Exploratory factor analyses

We explored the factor structure of each section of the PPSQ (parental playfulness, child playfulness, and perception and satisfaction with partner’s playfulness) using exploratory factors analyses (EFAs) with Promax rotation. In the parental playfulness section, we retained four factors, accounting for a total of 53.16% of variance: (a) playful effort, (b) teasing, (c) rule defiance, and (d) rough/tumble play (RTP) (see Table 2). The reliability was acceptable for the four factors in this parental section with Alpha scores ranging from 0.70 to 0.88. The correlation coefficients between factors ranged between *r* = 0.06, n.s. and *r* = 0.51, *p* < 0.001.

In the child playfulness section, we retained one factor, labeled rigidity/offended, accounting for a total of 51.66% of variance (see Table 2). The factor reliability was acceptable (⍺ = 0.68). In the perception and satisfaction with partner’s playfulness section, we retained three factors, accounting for a total of 68.31% of variance: (a) partner’s lack of effort, (b) partner’s recklessness, and (c) feeling of exclusion (see Table 3). The reliability was excellent for the first two factors (⍺ = 0.89 and 0.82 respectively), but weaker for the third factor (feeling of exclusion) which only included three items (⍺ = 0.60). This factor should also be interpreted with caution. The correlation coefficients between factors ranged between *r* = 0.20, *p* < 0.001, and *r* = 0.61, *p* < 0.001.

Finally, concerning the associations between the three dimensions assessed, the parent’s evaluation of their own playful effort was negatively and significantly associated with all three factors of the partner dimensions, *r* ranging from −0.17 and − 0.24, *p* < 0.01. Rule defiance was significantly and positively correlated with the perception of the partner being reckless (*r* = 0.23, *p* < 0.001) and feeling excluded (*r* = 0.23, *p* < 0.001). Finally, the perception of child rigidity was positively and significantly correlated with the perception of the partner’s lack of effort (*r* = 0.19, *p* < 0.001).

### Covariate analyses

The following variables were examined as potential covariates: age of the parent, sex of the parent, age of the child, parent education, child sex, and family gross income. Significant correlations were found for age of the parent, age of the child, parent education, and family gross income and various study variables (see Table 4). Subsequent analyses thus control for these variables. Of note, correlations were also identified between parent gender/child sex and study variables. However, given that some parents did not disclose their own and/or their child’s sex, the inclusion of these covariates would have lowered the number of participants significantly (*n* = 304 to *n* = 269). As such, parent gender and child sex were not included as a covariate.

**Table 4:** Correlation between PPSQ factors and potential control variables.

Controlled variables						
PPSQ factors	Age of parent	Sex of parent^a^	Age of child	Parent education	Child sex^b^	Family gross income
*Parent domain*						
Playful effort	−0.16**	0.04	−0.14**	−0.07	−0.01	−0.13*
Teasing	−0.14**	0.15**	−0.01	−0.15**	−0.06	−0.17**
Rule defiance	−0.06	0.14**	0.04	−0.07	0.06	−0.09
RTP	−0.11*	0.25***	−0.02	−0.05	0.13*	−0.10
*Child domain*						
Rigidity/offended	−0.01	0.01	−0.06	−0.06	−0.02	0.05
*partner domain*						
Partner’s lack of effort	0.01	−0.04	0.14*	−0.01	0.04	−0.04
Partner’s recklessness	−0.13*	−0.02	−0.01	−0.04	0.13*	−0.10
Feeling of exclusion	−0.06	0.07	−0.11	−0.06	0.04	−0.11

a 1 = mother; 2 = Father.

b 1 = girl; 2 = boy.

*p < 0.05, **p < 0.01, ***p < 0.001.

### Construct validity for the factor structure of the parental domain PPSQ section

In light of the factors that we obtained, we first explored the convergent validity through associations with similar factors measured with different questionnaires (e.g., RTP of PPSQ and RTP of CPBQ). As presented in Table 5, for the parental domain, we found the following associations: a strong correlation between the playful effort in the PPSQ and the playfulness total of the PPQ (*r_part._* = 0.61; *p* < 0.001) and a moderate correlation between the playful effort in the PPSQ and the playfulness total of the APS (*r_part._* = 0.43; *p* < 0.001). There was also a moderate correlation between teasing in the PPSQ and teasing of the CPBQ (*r_part._* = 0.39; *p* < 0.001) as well as a strong correlation between RTP of the PPSQ and RTP of the CPBQ (*r_part._* = 0.72; *p* < 0.001). Second, we assessed the predictive validity by exploring associations between concepts that are different but should be related (e.g., teasing of PPSQ and RTP of CPBQ). We found significant and positive correlations between the RTP score of the PPSQ and all other assessments (see Table 5). Interestingly, the RTP score of the CPBQ was also significantly and positively correlated to all PPQ scores (see Table 5).

**Table 5:** Partial correlations for the parent domain by controlling for parent age, child age, education, and income.

Related variables					
PPSQ: parental domain	Playfulness total (PPQ)	Playfulness total (APS)	Teasing (CPBQ)	RTP (CPBQ)	Challenge to risk (CPBQ)
Playful effort	0.61***	0.43***	0.01	0.27***	0.05
Teasing	0.14	0.15	0.39***	0.39***	0.14
Rule defiance	0.04	0.02	0.13	0.29***	0.02
RTP	0.22***	0.23***	0.22***	0.72***	0.23***
*M*	3.62	4.38	3.68	2.76	3.81
*SD*	0.45	0.73	1.22	0.83	0.73

**p < 0.01, ***p < 0.001.

As a follow-up to these analyses, we ran multiple regressions to explore which of these scores is a significant predictor of each parent factor of the PPSQ, when their shared variance was considered (see Table 6). Control variables were entered in the first step. The first regression revealed that the PPQ total score, the APS total score, and the CPBQ RTP score all significantly contributed to the prediction of the playful effort factor of the PPSQ. The second regression revealed that the CPBQ teasing score, and the CPBQ RTP score both significantly contributed to the prediction of the teasing factor of the PPSQ. The third regression revealed that only CPBQ RTP score significantly contributed to the prediction of the rule defiance factor of the PPSQ. Finally, the fourth regression revealed that CPBQ RTP score and CPBQ Challenge to risk score both significantly contributed to the prediction of the RTP factor of the PPSQ.

**Table 6:** Multiple regression models predicting factors from the parental domain.

	Δ*R^2^*	Δ*F*	*df*	*β*
	PPSQ – Parent domain: Playful effort			
Model	0.41	44.02***	5,298	
PPQ-total				0.51***
APS-total				0.18***
CPBQ-teasing				−0.02
CPBQ-RTP				0.14**
CPBQ-Risk				−0.08
	PPSQ – Parent domain: Teasing			
Model	0.25	20.87***	5,298	
PPQ-total				0.06
APS-total				0.04
CPBQ-teasing				0.33***
CPBQ-RTP				0.28***
CPBQ-risk				0.05
	PPSQ – Parent domain: Rule defiance			
Model	0.09	6.11***	5,298	
PPQ-total				−0.01
APS-total				−0.04
CPBQ-teasing				0.07
CPBQ-RTP				0.30***
CPBQ-Risk				−0.03
	PPSQ – Parent domain: RTP			
Model	0.53	70.04***	5,298	
PPQ-total				0.06
APS-total				0.06
CPBQ-teasing				0.06
CPBQ-RTP				0.71***
CPBQ-Risk				0.08*

Control variables (child age, parent age, education and income) were entered in a first step for each regression model.

*p < 0.05, **p < 0.01, ***p < 0.001.

### Construct validity for the factor structure of the child domain PPSQ section

Based on the obtained factor, we assessed predictive validity for the child domain through the associations of constructs that are different but should be related. However, we have not found any significant correlations between the rigidity/offended factor of the PPSQ and any of the other child playfulness measures. The follow-up multiple regression analyses confirmed this lack of association, as none of the other variables significantly predicted the PPSQ rigidity score (ΔR^2^ = 0.02, Δ*F* = 1.30, n.s.).

### Construct validity for the factor structure of the partner domain PPSQ section

As previously mentioned, there was a lack of questionnaires that assessed partner’s playfulness in the existing literature, preventing us from exploring convergent validity. As such, we explored predictive validity with constructs that are different but could be related to the ones of the PPSQ. As presented in Table 7, describing the partner domain of the PPSQ, we found a very similar pattern of association for the partner’s lack of effort factor and the partner’s recklessness factor of the PPSQ and different dimensions of the coparenting quality. Indeed, both factors show negative and significant moderate correlations with overall coparenting quality, agreement, support, partner parenting and division of labor (all *r_part._* Ranging between −0.21 and − 0.44; see Table 7). They were also moderately and positively associated with the undermining scale (*r_part._* = 0.34; *p* < 0.001 and *r_part._* = 0.42; *p* < 0.001, respectively). The feeling of exclusion factor of the PPSQ showed a similar pattern of association, although the associations with the support (*r_part._* = −0.19) and partner parenting (*r_part._* = −0.18) scales were only significant at *p* < 0.01. Only the closeness scale of the CRS was not associated with the PPSQ factors.

**Table 7:** Partial correlation for the partner domain by controlling for parent age, child age, education, and income.

Related variables							
PPSQ: partner domain	Coparenting quality (CRS)	Agreement (CRS)	Closeness (CRS)	Support (CRS)	Undermining (CRS)	Partner Parenting (CRS)	Div. Labor (CRS)
Partner’s lack of effort	−0.44***	−0.38***	−0.13	−0.21***	0.34***	−0.38***	−0.43***
Partner’s recklessness	−0.36***	−0.35***	−0.08	−0.23***	0.42***	−0.36***	−0.43***
Feeling of exclusion	−0.23***	−0.33***	−0.06	−0.19**	0.36***	−0.18**	−0.30***
M	5.00	4.11	5.18	5.33	2.72	5.05	3.60
SD	0.67	1.10	0.88	0.83	1.73	0.69	1.53

**p < 0.01, ***p < 0.001.

The first multiple regression analysis (see Table 8) revealed that the CRS partner parenting score, the CRS agreement score, and the CRS division of labor score all significantly and negatively contributed to the prediction of the perception of partner lack of effort factor of the PPSQ. The second regression revealed that the CRS undermining score, the CRS partner parenting score, and the CRS division of labor score all significantly contributed in the expected direction to the prediction of the perception of partner recklessness factor of the PPSQ. Finally, the third regression revealed that the CRS agreement score, the CRS undermining score, and the CRS division of labor score all significantly contributed in the expected direction to the prediction of the perception of feeling of exclusion factor of the PPSQ.

**Table 8:** Multiple regression models predicting factors from the partner domain.

	Δ*R^2^*	Δ*F*	*df*	*β*
	PPSQ – Partner domain: Lack of effort			
Model	0.30	15.26***	7,248	
CRS-Total				−0.20
CRS-agreement				−0.14*
CRS-closeness				0.09
CRS-support				0.04
CRS-undermining				0.08
CRS-parenting				−0.21***
CRS-division				−0.18*
	PPSQ – Partner domain: Recklessness			
Model	0.30	15.80***	7,248	
CRS-total				0.05
CRS-agreement				−0.12
CRS-closeness				0.07
CRS-support				−0.08
CRS-undermining				0.20**
CRS-parenting				−0.19***
CRS-division				−0.26***
	PPSQ – Partner domain: Exclusion			
Model	0.19	8.66***	7,248	
CRS-total				0.23
CRS-agreement				−0.21**
CRS-closeness				−0.03
CRS-support				−0.15
CRS-undermining				0.20**
CRS-parenting				−0.04
CRS-division				−0.23**

Control variables (child age, parent age, education, and income) were entered in a first step for each regression model.

* *p* < 0.05, ** *p* < 0.01, *** *p* < 0.001.

## Discussion

In line with recent research seeking to investigate under-explored types of positive parenting behaviors that may contribute to children’s wellbeing and positive child–parent relationships (Cabrera and Roggman, 2017; Menashe-Grinberg and Atzaba-Poria, 2017; Atzaba-Poria, 2018), this study examined the psychometric properties of a new questionnaire on playful parenting encompassing dimensions related to the parent’s own playfulness, child playfulness, and perception of partner’s playfulness. The findings suggest that the PPSQ is a valid measure of parental playfulness and perception of partner’s playfulness, given its association with similar and related measures. However, we failed to show association for our child playfulness factor.

### Factor structure of the PPSQ

An exploratory factor analysis revealed different factors for the three domains assessed. The factor solution for parent’s own playfulness revealed four dimensions: Playful effort, Teasing, Rule defiance, and Rough and tumble play (RTP). Analyses also revealed a Rigidity/offended dimensions for child playfulness. Finally, three factors were found for the partner domain: Partner’s lack of effort, Partner’s recklessness, and Feeling of exclusion. It should be noted that the later factors (Feeling of exclusion) did not show adequate reliability, possibly due to an insufficient number of items. It would be important to consider adding similar items in a future revision of the questionnaire to explore whether this factor could reach a stronger reliability. Overall, the factors obtained for the parent domain are coherent with other instruments that revealed concepts such as general playfulness or playfulness effort (Shorer et al., 2021), RTP, and teasing (Majdandžić et al., 2016). For the child domain, it was rather surprising that no factor relating to child playfulness in general was found, as observed in other studies (Barnett, 1991; Rogers et al., 1998; Trevlas et al., 2003). However, most instruments rely on parents’ observation of child play with peers (see Trevlas et al., 2003), which is a specific context that may differ from child playfulness in general (i.e., in their daily routine). It was also surprising that the only emerging factors were child rigidity and being easily offended, although it is reasonable to interpret lower scores on this scale (i.e., child is not rigid and takes jokes lightly) as a marker of playfulness. Finally, no comparable instrument assessed a partner’s playfulness, preventing us from exploring the convergent validity of this part of the questionnaire.

As part of our exploration of the internal structure of the PPSQ, we tested the correlations between the factors included in the three dimensions of the instrument. Results revealed interesting associations supporting our rationale for creating an instrument encompassing the perception of different family members’ playfulness under a family system framework (Kerr, 1981). Indeed, parents who perceived themselves as more playful also rated their partners as making greater effort to play with their child, being less reckless and feeling less excluded from their relationship with their child. These associations may result from a positive bias by the reporting parent, who views themselves and their partner more favorably. However, it may also indicate that adults with similar values and personalities are more likely to form a couple and have children together, a phenomenon identified as assortative mating (Schwartz, 2013). The fact that parents who rate themselves as promoting rule defiance also perceive their partner as being more reckless would be coherent with such an explanation. Also interesting is the positive association between the perception of child rigidity and the partner’s lack of effort. Does it reflect some form of disappointment in both the child and partner regarding a lack of playfulness? Further research is needed to explore these hypotheses.

### Construct validity of the PPSQ: parent domain

A second goal of this study was to explore the construct validity of the PPSQ factors through convergent and predictive validity. We examined the convergent validity of the factors related to parent’s own playfulness in relation to questionnaires assessing overall playfulness, teasing, and RTP (i.e., questionnaires corresponding to similar factors revealed in the PPSQ). We found that the playfulness effort and RTP dimensions of the PPSQ were highly correlated with the total score of playfulness on the PPQ and the RTP dimension of the CPBQ, respectively. This suggests good construct validity for these dimensions. Further, we found that there was a significant but moderate correlation between the teasing dimensions of the PPSQ and the CPBQ, suggesting acceptable construct validity. All of these associations were further supported by the multiple regression analyses showing that the playful score of the PPT, the RTP score of the CPBQ, and the teasing score of the CPBQ were the strongest predictors of the playful score, the RTP score, and the teasing score of the PPSQ, respectively.

As part of this objective, we also assessed the predictive validity of the PPSQ by examining the associations between concepts that are different but should nonetheless be related. For the parent domain, we observed a positive but moderate correlation between the playful effort of the PPSQ along with the adult playfulness of the APS. This association was also significant in the regression analyses. We also found moderate positive associations between the RTP scale of the CPBQ and all other assessment scales (playfulness, teasing, and challenge to risk). Once again, the regression analyses showed that the RTP scale of the CPBQ remained a significant predictor of all of these PPSQ scales even when shared variance with other variables was controlled for. Lastly, the playful, teasing and rule defiance scales of the PPSQ were positively and moderately related to the RTP scale of the CPBQ, although the teasing score was not a significant predictor in the regression model. The moderate association between the playful effort and adult playfulness in general may suggest that trait playfulness is partially independent from playfulness in the child–parent dyad. In their definition of adult playfulness, Glynn and Webster (1992) insist on a trait-like nature of playfulness “i.e., a predisposition to define and engage in activities in a nonserious or fanciful manner to increase enjoyment” (p. 83). This is in line with Barnett’s (1991) conceptualization of playfulness as an individual predisposition. In comparison, in their definition of parental playfulness, Menashe-Grinberg and Atzaba-Poria (2017) and Cabrera et al. (2017) describe parental behavioral patterns specific to the particular context of the child–parent interaction, without generalizing it to other domains of the parent’s life. The moderate association found in our study indeed suggests that parental playfulness may be domain-specific, at least for some individuals. Some parents may be more comfortable being playful (e.g., acting silly) with their children than with other adults, or perceive the importance of creativity, joy, imagination, and humor in their child’s development, and therefore may act more playfully when interacting with their children. In future research, it would be interesting to merge these previously mentioned constructs of playfulness to exemplify a biopsychosocial perspective toward parental playfulness.

The modest but significant association between various dimensions of playfulness and RTP also suggests that although RTP is not identical to playfulness in general, it seems to be one of its central dimensions. Indeed, RTP, being assessed with the PPSQ or the CPBQ, is the only concept that is significantly related to all other scores. In fact, considering the nature of interactions in the preschool and early school years, it may not be surprising that RTP plays an important role with young children. For example, an observational study by Schmiedel (2021) revealed that fathers and mothers use chasing and tickling as the most frequent method to make their preschool children laugh in a Laughing Task procedure (Bureau et al., 2014, 2017). Schmiedel (2021) also observed that physical methods were more efficient to make children laugh as compared to more verbal (e.g., telling jokes or funny stories) or solitary methods (e.g., clowning, funny faces). Although they did not explore the association between independent markers of RTP and playful effort in their study, Majdandžić et al. (2016) instead embedded the effort deployed by the parent as part of their observed RTP scale, suggesting that they conceptualize these two concepts as being highly related. For example, a parent who quietly chases a child would score low on their RTP scale, whereas moderately intense tickling would get a medium score, and frightening a child for fun would result in a high score. Although these behaviors are conceptualized by Majdandžić et al. (2016) as indicators of challenging parenting behaviors, they are also most likely reflective of the parent’s effort to be playful. The similarities between the concepts of RTP and physical play in the literature are also discussed in a meta-analysis on RTP by StGeorge and Freeman (2017). Unfortunately, other studies exploring playfulness more specifically as a stand-alone construct did not take into account the association between this concept and RTP (Atzaba-Poria et al., 2014; Majdandžić et al., 2016; Cabrera et al., 2017; Aldoney and Prieto, 2021; McDorman et al., 2021).

Despite the limited evidence supporting an association between playful effort and physical RTP in younger children, Bureau et al. (2021) noted that the physical component is far less prominent when parents are trying to make children laugh in middle childhood (8–11-year-olds) compared to the preschool years. When children are older, parents would rather tell jokes or dare the child not to laugh while making funny faces. This is in line with the suggestion that challenging behaviors may become more cognitive in nature over time. Therefore, one may expect that the interchangeable nature of physical play and RTP noted by StGeorge and Freeman (2017) may be more specific to the infancy period, and that these concepts may be more easily distinguishable at later ages, when play takes different forms, not necessarily of a physical nature. Future studies are needed to explore how physical play may change over different developmental periods and examine whether physical RTP is a more prominent component of playfulness in early childhood.

### Predictive validity of the PPSQ: child domain

Concerning the child domain, unfortunately, we did not obtain factors matching those from previous measures, making the comparison less straightforward. However, the PPSQ factors seem to capture a certain lack of spontaneity in children (rigidity/easily offended) that is also at the heart of Barnett’s instrument Barnett’s (1991) which deals with different types of spontaneity in children, or lack thereof, as reported by their parent (cognitive, social, physical spontaneity). However, we failed to find a significant negative association between the PPSQ dimension of rigidity/offended and social spontaneity on the CPS (Barnett, 1991).

Overall, our conceptualization of child playfulness aligns with the definition provided by Trevlas et al. (2003), which defines child playfulness as an internal predisposition to bring a playful quality to interactions within the environment and across a variety of contexts. However, most previous research assessed playfulness in the context of observations or observation scales that focused on frequency counts of child’s play with peers (Trevlas et al., 2003). This only represents one particular context that may not generalize to daily child–parent interactions. This could partially explain the general discrepancy between our results and those from previous studies. Based on current results, it appears important to revise extensively the items of the PPSQ pertaining to child domain if we aim at aligning our concepts with those assessed in previous measures. Alternatively, we may further explore how the Rigidity/offended factor may be associated to child outcomes. It is possible that it captures playfulness as an internal predisposition as opposed to a contextual variable observed with a specific group of peers.

### Predictive validity of the PPSQ: partner domain

We explored the association between this domain and a closely related construct, that is the quality of the coparenting relationship. As expected, the factors pertaining to the partner’s domain (i.e., partner’s lack of effort, partner’s recklessness, and feeling of exclusion) were generally significantly correlated with the quality of co-parenting, in the expected direction. The multiple regression analyses further confirmed that the parent’s perception of the partner as not making enough effort to play with their child was significantly predicted by a more negative perception of this partner’s parenting skills, a lower agreement between parents, and a lower satisfaction with the division of labor. The lower satisfaction with division of labor and the more negative perception of partner parenting were also significant predictors of the perception of the partner being perceived as reckless when playing with their child, along with more undermining between parents. Finally, parents’ report of lower agreement, more undermining, and less satisfying division of labor all significantly predicted their feeling of exclusion on the PPSQ. The fact that a negative appraisal of a partner’s playfulness was associated with so many dimensions of coparenting in the CRS (Feinberg et al., 2012) may suggest that it is part of a more general concept of coparenting quality that has been overlooked in past research. Whereas a disagreement about the type of parental discipline to be used or the autonomy allowance may seriously affect the coparenting relationship in general, it is also possible that when a partner makes no effort to be playful with their child or uses sarcasm and teasing when the other parent does not value these forms of interaction, it may become a source of conflict between parents. Indeed, the challenging and disruptive nature of playful parenting may not be well received by the other parent. It has been shown in previous studies that a disagreement between parents may result in less playful behaviors (McBride et al., 2005; Andersen et al., 2017). However, our study suggests that it is not only reckless behavior that may foster some resistance in the other parent, but also a general lack of effort to play with the child. Taken together, these results suggest that a right dosage (not too low nor too high) of playfulness may be seen as optimal by a partner. It is also interesting to note that the feeling of exclusion was also associated with various dimensions of coparenting despite that this factor is of a slightly different nature than the other two previously mentioned. Indeed, whereas the other two factors involve an evaluation of the quality of the partner’s behaviors, the feeling of exclusion factor assesses how the parent feels about the child-partner relationship, regardless of the partner’s behavior. For example, one may think that a parent is more likely to feel excluded when they perceive their partner as more competent and able to establish a special connection with the child. In line with this suggestion, the feeling of exclusion was the only factor among the three that was not predicted by a lower perception of the partner’s parenting skills. It was rather predicted by variables indicative of disagreement between parents.

### Limitations

While this study supports the validity of the PPSQ, it is not without limitations. First, although the sample size is quite large, it does not allow us to explore more specific questions such as the effect of parent gender and child sex. The father group is notably limited in size, preventing us from running robust comparisons between mothers and fathers. Second, the study is based on a convenience sample, which may impact the generalizability of the findings. It is possible that parents who volunteer to participate in such a study on playfulness are more involved in parenting in general and/or may consider themselves as being more playful. Third, the study was conducted during the COVID-19 pandemic, and it is unclear if and how the pandemic would influence parents’ responses to questionnaires. Fourth, the sample is quite homogeneous in terms of ethnicity and socioeconomic status (SES). Future studies with more diverse samples are needed to better understand the concept of parental playfulness, which may vary across contexts. Fifth, this study is the first to assess a partner’s playfulness, which prevented us from comparing this section of the PPSQ to existing measures of partner’s playfulness. Sixth, the current study does not involve a test–retest design, does not include observations of parent or child playfulness which would counter-balance a potential social desirability bias in the questionnaire, and does not include a confirmatory analysis of the factors in a separate sample. Finally, the present study does not address the connection between playfulness and children’s cognitive or social development. All these limitations will need to be addressed in future studies in order to further establish the validity and usefulness of the PPSQ.

## Conclusion

This study allowed for a better understanding of the playful dynamics that occur within a family, via the PPSQ, by taking into account different agents such as a parent, a child, and a partner. Our results support the construct and convergent validity in respect to parental playfulness and the perception of the partner’s playfulness due to its association with similar and related measures. It was not possible, however, to determine this with the child’s playfulness factor. Beyond constructs such as parental sensitivity, parental playfulness may be indicative of a parent who enjoys interacting with their child and values this relationship, which could contribute to child wellbeing beyond other parental behaviors. Previous research has shown that parental playfulness has a positive effect on a child’s socio-emotional development. Our study tested the psychometric properties of a more comprehensive assessment of playfulness among family members (parents, children, and partners), which will allow for increased specificity in understanding the benefits of playfulness in the family.

## Data availability statement

The raw data supporting the conclusions of this article will be made available by the authors, without undue reservation.

## Ethics statement

The studies involving humans were approved by the University of Ottawa Research Ethic Board. The studies were conducted in accordance with the local legislation and institutional requirements. The participants provided their written informed consent to participate in this study.

## Author contributions

J-FB: Conceptualization, Formal analysis, Funding acquisition, Investigation, Methodology, Project administration, Supervision, Writing – original draft, Writing – review & editing. KB: Formal analysis, Writing – original draft, Writing – review & editing. A-AD: Conceptualization, Investigation, Methodology, Writing – review & editing. JT: Writing – review & editing. HS: Data curation, Investigation, Writing – review & editing. PB-L: Formal analysis, Funding acquisition, Investigation, Writing – review & editing.
